# Gastric cancer surgery: Billroth I or Billroth II for distal gastrectomy?

**DOI:** 10.1186/1471-2407-9-428

**Published:** 2009-12-09

**Authors:** Birendra K Sah, Ming-Min Chen, Min Yan, Zheng-Gang Zhu

**Affiliations:** 1 Department of General Surgery, Rui Jin Hospital, Shanghai Jiao Tong University, School of Medicine, Shanghai Institute of Digestive Surgery, Shanghai, China

## Abstract

**Background:**

The selection of an anastomosis method after a distal gastrectomy is a highly debatable topic; however, the available documentation lacks the necessary research based on a comparison of early postoperative complications. This study was conducted to investigate the difference of early postoperative complications between Billroth I and Billroth II types of anastomosis for distal gastrectomies.

**Methods:**

A total of 809 patients who underwent distal gastrectomies for gastric cancer during four years were included in the study. The only study endpoint was analysis of in-patients' postoperative complications. The risk adjusted complication rate was compared by POSSUM (Physiological and operative severity score for enumeration of morbidity and mortality) and the severity of complications was compared by Rui Jin Hospital classification of complication.

**Results:**

Complication rate of Billroth II type of anastomosis was almost double of that in Billroth I (P = 0.000). Similarly, the risk adjusted complication rate was also higher in Billroth II group. More severe complications were observed and the postoperative duration was significantly longer in Billroth II type (P = 0.000). Overall expenditure was significantly higher in Billroth II type (P = 0.000).

**Conclusion:**

Billroth II method of anastomosis was associated with higher rate of early postoperative complications. Therefore, we conclude that the Billroth I method should be the first choice after a distal gastrectomy as long as the anatomic and oncological environment of an individual patient allows us to perform it. However more prospective studies should be designed to compare the overall surgical outcomes of both anastomosis methods.

## Background

In the surgical approach for early and selective advanced gastric cancer, the gastrectomy with D2 lymphadenectomy was justified [1-5]. However the surgery procedures for gastric cancer vary from one to another unit. The extent of surgery for gastric cancer is highly heterogeneous. Certainly there are differences in morbidity rates associated with the different extents of surgery, even though they are all commonly denoted as radical resection [6-12]. Though postoperative complications and the mortality rate after gastric cancer surgery has significantly decreased over past years, it is still considered high [13,14]. The postoperative complication rate was higher with inexperienced surgeons than with experienced surgeons, and there was a considerable difference in early surgical outcomes among different centers [15,16]. Postoperative complications were inversely correlated with the volume of patients operated on in a surgical unit [17]; the same results were published for gastric cancer surgery [18].

There are many controversies over gastric cancer surgery, but there are comparatively fewer articles which are dedicated to early postoperative complications of gastric cancer surgery [14,18,19]. Therefore research on early postoperative complications may be beneficial to give reference points that can help to optimize the success of gastric cancer surgery.

There were reports of the comparison of different types of the anastomosis method after a gastrectomy, however, these reports were basically focused on investigating the bile or enteric juice reflux into the gastric remnant and esophagus following a gastrectomy. The majority of these reports advocated for Roux-en-Y Reconstruction and some for Billroth I (gastroduodenostomy) method of anastomosis. Billroth II (gastro-jejunostomy) method was not supported by the results of these tests [20-26].

In our clinical practice we observed that postoperative complications were higher in the Billroth II type of anastomosis, therefore we compared the early postoperative complications of patients with the Billroth I and Billroth II type of anastomosis. The POSSUM (physiological and operative severity score for the enumeration of morbidity and mortality) scoring system [27] was applied for the risk adjusted comparison of early postoperative complications between two groups. The POSSUM system was a valid system to evaluate the risk adjusted comparison of surgical outcomes in gastric cancer surgery [18,19].

First described in 1991 by Copeland et al, POSSUM was developed as an attempt to assess the quality of surgical care [27]. This scoring system produced assessments for morbidity and mortality rates which did not significantly differ from observed rates. For the development of POSSUM system, initially 62 individual factors were assessed by a multivariate discriminant analysis to reduce the number of variables. Finally, a 12-factor physiological score(Age group, Cardiac status, ECG report, Respiratory status, Systolic blood pressure, Pulse rate, Glasgow coma scale, Hemoglobin, White cell count, Urea, Sodium, Potassium) and a 6-factor operative severity score (Operative complexity, Multiple procedures, Blood loss, Peritoneal contamination, Extent of malignant spread, Elective or emergency surgery) were developed. Each of the factors were graded and scored exponentially as 1, 2, 4 or 8. Logistic regression analysis yielded statistically significant equations for morbidity. Although the higher the overall POSSUM score, the greater the risk of morbidity and mortality, individual scores do not directly reflect the percentage risk [27].

## Methods

The data were collected directly by the comprehensive review of the original records of all patients. A total of 809 patients who underwent radical or palliative distal gastrectomies for gastric cancer during four years was included in the study (Table 1). Any pathology other than that of the gastric cancer was excluded. Total gastrectomies and any type of palliative surgery (including exploratory laparotomy and gastro-jejunal anastomosis) other than gastrectomy were excluded. The median age of the patients was 58 years (range 17-88 years). All the patients with early and resectable advanced gastric cancer (without significant distant metastases) underwent radical surgery (gastrectomies with D2 lymphadenectomy). Late stage gastric cancer patients underwent a palliative gastrectomy. Because of inadequate numbers of examined lymph nodes, we could not document all the pathological data according to the TNM classification.

**Table 1:** Demographic data of the patients

Details		Billroth I	Billroth II
Number of patients		626	183
Age group (years)	≤60	355	100
	61-70	146	39
	≥71	125	44
Sex	Male	394	127
	Female	232	56
Type of resection	Radical gastrectomy	599	160
	Palliative gastrectomy	27	23
Malignancy	Primary tumor	323	83
	Lymph node metastasis	292	97
	Distant metastasis	11	3
Automatic stapler	No	403	131
Median value	Yes	221	52
	PS	15	15
	OSS	18	18

* Including recurrent gastric cancer.

The only study endpoint was the analysis of in-patients' postoperative complications. Complications were recorded according to the definitions mentioned in POSSUM [27]. However, there were a considerable number of complications which were not covered by its definitions. Therefore any undefined complication was recorded as "innominate" in this study, and details were provided in separate tables. Severities of all complications were stratified according to Rui Jin Hospital Classification of Complication [14,18,19]. Patients with multiple complications were grouped into the highest level of their respective complications, e.g. a patient with minor, moderate, and severe complications was categorized into the severe complication group (Table 2).

**Table 2:** Rui Jin Hospital classification of complications

**Minor**	**Infection**: Superficial wound infection, deep infection*, chest infection*, urinary infection, septicemia, pyrexia of unknown origin* Miscellaneous: Superficial wound dehiscence, wound hemorrhage, impaired renal function*, deep venous thrombosis *, Hypotension.
**Moderate**	**Infection**: Deep infection^†^, chest infection^†^, pyrexia of unknown origin^†^. **Miscellaneous**: deep wound dehiscence, impaired renal function ^†^, deep venous thrombosis ^†^ **Innominate^†^**
**Severe**	**Systemic**: Cardiac failure, Respiratory failure, pulmonary embolus, Hypotension^‡^. Death **Surgical**: Deep hemorrhage, deep infection^‡^, anastomotic leak **Innominate**: complications with postop stay >30 days

*(postop ≤ 15 days), † (postop > 15 days), ‡ (Requiring laparotomy)

Except "innominate", the definition for all complications was adopted from POSSUM.

### Statistical analysis

The statistical analysis was performed with the Statistical Package for Social Science (SPSS) version 13.0 for Windows (SPSS, Inc, Chicago, Illinois). A chi square test was used to compare the different types of complication rates between two groups. Non-parametric methods were used to test the data without normal distribution. A P-value of less than 0.05 was considered statistically significant.

### Formula for risk calculation in POSSUM

Morbidity: ln R/1-R = -5.91 + (0.16 × PS) + (0.19 × OSS)

Where "R" is predicted risk. PS and OSS stands for physiological score and operative severity score respectively.

The exponential analysis method was used for prediction of morbidity rate [27]. After calculation of predicted morbidity, the observed-to-predicted operative morbidity ratio (O: E ratio) was calculated separately for Billroth I and Billroth II group. An O: E ratio less than one implies a performance that was better than expected, and a ratio greater than one indicates a performance that was worse than expected [18,19].

## Results

Details of complications according to the POSSUM criteria were summarized in table 3. The sum of the individual complications was not equal to the number of total complications. Multiple complications were possible in a single patient. There was a significant difference in the complication rate between groups of patients with Billroth I and Billroth II types of anastomosis (P = 0.000). The complication rate of the Billroth II type of anastomosis was almost double of that in Billroth I. Incidence of different types of postoperative infection was significantly higher in the Billroth II type. The anastomotic leak and mortality rate were also higher in the Billroth II type, though there was no statistically significant difference in those observations.

There was no significant difference of malignancy status between Billroth I and Billroth II group (p = 0.316). But the complication rate was significantly higher in Billroth II than Billroth I group even after controlling the malignancy status (p < 0.001). To control the effect of different type of resection on postoperative complication, we calculated the complication rate separately for radical and palliative resection. The complication rate of Billroth II was significantly higher than Billroth I in group of patients who underwent standard radical gastrectomy but not in the patients who underwent palliative gastrectomy (table 4).

**Table 3:** Detail of complications

Complications		Billroth I	Billroth II	P value
Overall complication		126(20.1)	68(37.2)	0.000
Hemorrhage	Deep	3(0.5)	2(1.1)	NS
Wound dehiscence	Superficial	2(0.3)	2(1.1)	NS
	Deep	4	0	NS
Anastomotic leak		8(1.3)	6(3.3)	NS
Infection	Wound	2(0.3)	2(1.1)	NS
	Deep	17(2.7)	19(10.4)	0.000
	PUO*	82(13.1)	36(19.7)	0.027
	Chest	17(2.7)	15(8.2)	0.001
	UTI^†^	2(0.3)	3(1.6)	NS
	Multiple	10(1.6)	10(5.5)	0.003
System failure	Renal	7(1.1)	4(2.2)	NS
	Respiratory	2(0.3)	2(1.1)	NS
	Cardiac	2(0.3)	1(0.5)	NS
	Hypotension	2(0.3)	1(0.5)	NS
DVT^‡^		0	1(0.5)	NS
Death		1(0.2)	2(1.1)	NS

*pyrexia of unknown origin, † urinary tract infection, ‡ deep venous thrombosis

**Table 4:** Complication rate between Billroth I and Billroth II reconstruction

		Complication		P value
			
Resection	Anastomosis	No	Yes	
Radical	Billroth I	483(80.6)	116(19.4)	< 0.001
	Billroth II	99(61.9)	61(38.1)	
Palliative	Billroth I	17(63.0)	10(37.0)	NS
	Billroth II	16(69.6)	7(30.4)	

Moreover, the ratio of observed to estimated complication was 1.03 for the Billroth II group while it was only 0.74 in the Billroth I. This revealed that the surgical outcome was poorer in the Billroth II group.

There were numerous "innominate" complications, and most of these complications were accompanied by complications described in POSSUM (table 5). The majority of patients had pleural effusion and/or seroperitoneum. Most of them were accompanied by a low fever but lacked a pathological diagnosis of infections. There were a substantial number of patients who had a persistent or relapsing fever of unknown origin. There were a number of patients who were clinically suspected to have a minor anastomotic leak but lacked any objective evidence to support it. Though these patients were settled by conservative treatments mainly nil per os, intravenous antibiotics and total paraenteral nutrition, these cases obviously increased the burden of the surgical ward. Other complications like pancreatic fistula, chyle leak and bleeding of the anastomosis site were rare. Almost all types of innominate complications were also higher in the Billroth II type.

**Table 5:** Innominate complications

Complications	Billroth I	Billroth II	P value
Pleural effusion	26(4.4)	14(7.7)	NS
Consistent fever of unknown reason	55(8.8)	26(14.2)	0.032
Seroperitoneum	27(4.3)	22(12.0)	0.000
Gastro or enteroplegia	19(3.0)	14(7.7)	0.005
Pancreatitis	1(0.2)	9(4.9)	0.000
Central vein catheter infection	6(1.0)	2(1.1)	NS
Anastomosis site or upper GI bleeding	1(0.2)	4(2.2)	0.010
Chyle leak	1(0.2)	1(0.5)	NS
Pancreatic fistula	0	1(0.5)	NS

**Figure 1:**
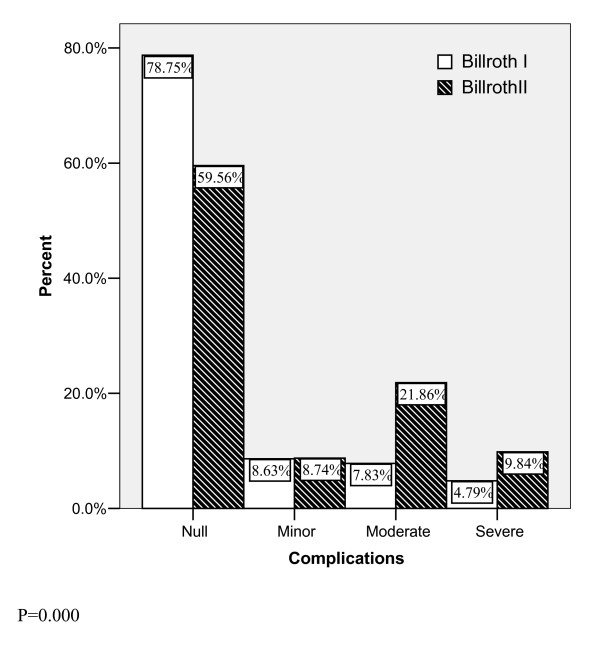
**Severity level of overall complications**.

Among cases of innominate complications, some patients did not experience any complications described in POSSUM. These complications were recorded empirically and merged to calculate different levels of complication type according to Rui Jin Hospital classification of complications. There was significant difference in severity of complications between the two groups (P = 0.000). More severe complications were observed in the Billroth II type (fig. 1).

**Figure 2:**
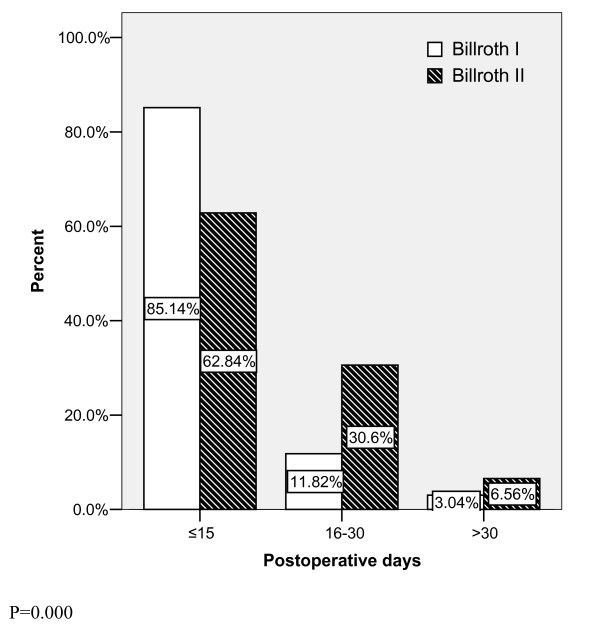
**Postoperative duration at hospital**.

All of the patients were categorized into three levels according to their postoperative stay at the hospital. There were significant differences in the postoperative duration between two groups of patients (P = 0.000). About 85 percent of patients were discharged successfully in less than 15 days after a smooth recovery and removal of suture in the Billroth I type (fig. 2). The postoperative duration was significantly longer in the Billroth II type (P = 0.000).

**Figure 3:**
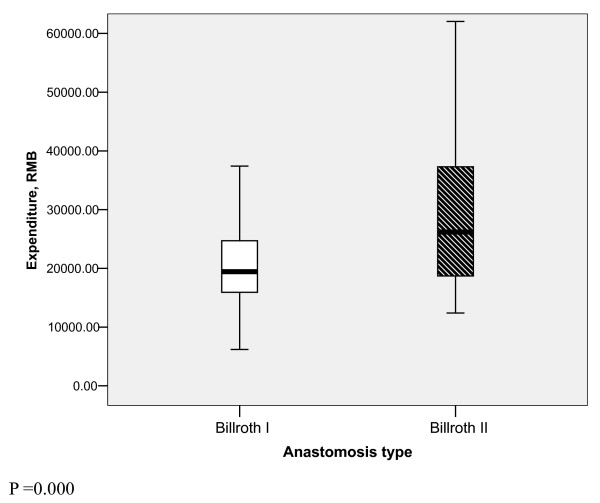
**Difference of expenditure between two groups**.

Similarly, there was a significant difference in overall expenditure between the two types of anastomosis method (P = 0.000). The median expenditure value in patients with Billroth II was 26175.26 RMB (Chinese currency) but it was only 19438.82 RMB for Billroth I (fig. 3)

## Discussion

The comparison of surgical outcome is made more difficult due to a lack of standard definitions for complications and reliable auditing methods. Simply collecting outcome data alone is not sufficient to reflect treatment quality; because to compare postoperative complication data directly, the original populations must be identical. POSSUM has been proposed as a method for standardizing patient data, so direct comparisons of surgical outcomes can be made. However there are a number of complications (e.g., pancreatic or biliary leakage, chylus leakage, ileus, enteroplegia and pulmonary complications) that are not defined in POSSUM [18,19]. Although some of these complications seem to be minor, they can markedly extend the postoperative length of stay and treatment cost and should not be ignored. Therefore POSSUM may need some modifications to be appropriate for major surgical interventions.

It was a well accepted truth that the extent of surgery, especially aggressive lymph node dissection, was useless to extend overall survival. Postoperative complications were significantly related with the extent of surgery, especially with the extent of lymph node dissection. This was even proven by Japanese surgeons who reported in the New England Journal of Medicine [15,28-30]. Therefore, gastric cancer surgery should be practiced only at experienced centers, and the extent of surgeries or procedures should be tailored to the competence of surgeons in this field [18]. It is imperative for non-specialized units to follow the experience of specialized units for gastric cancer surgery.

The postoperative complication rate of our hospital was in the acceptable range. A majority of our patients had a smooth recovery, and postoperative mortality was not higher than previously reported data. Our hospital, which deals with a high work volume, is a referral center for gastric cancer in China. Our hospital prefers the Billroth I method of anastomosis over the Billroth II method, and we seldom use the Roux-en-Y method after a distal gastrectomy.

Our study suggests that the early postoperative complication rate is significantly lower in Billroth I group. Despite the fact that there was not standard follow-up data, the operative surgeons in our center feel that the long term patients' satisfaction is also better with the Billroth I method. The Billroth I procedure is generally simpler to perform than other methods. It is considered to be more physiologically sound by preserving the continuity of the digestive tract with the duodenum and theoretically maintaining autocrine and paracrine signaling and feedback mechanisms [31-34]. Therefore the Billroth I type of anastomosis is superior to Billroth II.

The major hurdle in selecting the Billroth I method is the anatomic and oncological environment of the tumor. If the tumor is more advanced and the location is more distal to the duodenum, it is difficult to perform the Billroth I anastomosis. This is because it is difficult to obtain tumor-free margins, and it may further increase the tension between anastomosis edges which may be associated with higher anastomotic leak.

Interestingly enough, a higher rate of anastomotic leak was observed in the Billroth II group of this study. The overall complication rate was higher in the Billroth II group, especially the infectious complications. Intra-abdominal infection was also higher in the Billroth II group which may be the result of higher rate of postoperative pancreatitis and anastomotic leak. It may not be that the infectious complications were simply caused by pancreatitis or anastomotic leakage, but certainly higher the surgical insult or complexity of a surgery the higher will be risk of having more complication rate including the infectious complication rate. We trust the lower complication rate in Billroth I group may be attributed to its simplicity in terms of surgical insult and elimination of duodenal remnant which is very prone to rupture in case of distal obstruction or ileus. Also the early rupture of duodenal remnant induces peripancreatic abscess or intra-abdominal infection. However a well controlled prospective study is necessary to explore the potential factors behind the higher rate of infectious complications and anastomotic leaks. One can also argue that the difference in complication rate is simply the result of the inadequate surgical experience of the surgeon who prefers Billroth I against Billroth II, and better surgical outcome could be achieved in centers where the Billroth II is a preferred method. Though it was quite difficult to obtain a concrete answer for the choice of which anastomosis method should be preferred, the findings of this study at the very least demand further investigation into the potential causes of differences in surgical outcomes between the two different types of anastomosis. This necessitates integrative research to compare both the early and long-term aspects of the different anastomosis methods.

If the early postoperative outcome is better with the Billroth I method, it should be preferred for its simplicity and better physiological reconstruction. However the long-term patients' satisfaction should also be taken into consideration, and operative surgeons should tailor the appropriate method in accordance with the prognosis of the tumor.

The Roux-en-Y method of anastomosis was advocated by western authors [20-26] where the surgical work load was significantly lower than eastern countries like China, Korea and Japan. This method is not preferred in eastern countries including our center due to its complexity and lengthiness in comparison to the Billroth I method. Because of a higher rate of incidence of gastric cancer in the region, surgeons of a big hospital in eastern countries have to deal with a huge surgical work volume [14,18]. Therefore it is wise to explore the simpler surgical methods for gastric cancer surgery which benefit both surgeons and patients.

## Conclusion

The overall postoperative complication is still higher after gastric cancer surgery, though the severe complications and mortality rate are lower. The Billroth II method of anastomosis is associated with a higher rate of early postoperative complications. Therefore we conclude that the Billroth I method should be the first choice after a distal gastrectomy as long as the anatomic and oncological environment of the individual patient allows it. However more prospective studies should be designed to compare the overall surgical outcomes of both anastomosis methods.

## Competing interests

The authors declare that they have no competing interests.

## Authors' contributions

BKS designed the study, collected the patients' data and drafted the manuscript. CMM, YM and ZZG participated in the design of the study, assisted in drafting of the manuscript and performed the critical revision. All authors meet the criteria; they have read and approved the final manuscript.

## Appendix

### Definitions ofmorbidity in POSSUM system by Copeland et al

1. Hemorrhage: Wound hemorrhage: local haematoma requiring evacuation. Deep hemorrhage: postoperative bleeding requiring re-exploration.

2. Chest infection: production of purulent sputum with positive bacteriological cultures, with or without chest radiography changes or pyrexia, or consolidation seen on chest radiograph.

3. Wound infection: wound cellulitis or the discharge of purulent exudates.

4. Urinary infection: the presence of > 10 ^5 ^bacteria/ml with the presence of white cells in the urine, in previously clear urine.

5. Deep infection: the presence of an intra-abdominal collection confirmed clinically or radiologically.

6. Septicemia: positive blood culture.

7. Pyrexia of unknown origin: any temperature above 37°C for more than 24 h occurring after the original pyrexia following surgery (if present) had settled, for which no obvious cause could be found

8. Wound dehiscence: superficial or deep wound breakdown.

9. Deep venous thrombosis and pulmonary embolus: when suspected, confirmed radiologically by venography or ventilation/perfusion scanning or diagnosed at post mortem.

10. Cardiac failure: symptoms or signs of left ventricular or congestive cardiac failure which required an alteration from preoperative therapeutic measures.

11. Impaired renal function: arbitrarily defined as an increase in blood urea of > 5 mmol/l from preoperative levels.

12. Hypotension: a fall in systolic blood pressure below 90 mmHg for more than 2 H as determined by sphygmomanometer or arterial pressure transducer measurement.

13. Respiratory failure: respiratory difficulty requiring emergency ventilation.

14. Anastomotic leak: discharge of bowel content via the drain, wound or abnormal orifice.

## Pre-publication history

The pre-publication history for this paper can be accessed here:

http://www.biomedcentral.com/1471-2407/9/428/prepub
